# CD73‐generated extracellular adenosine promotes resolution of neutrophil‐mediated tissue injury and restrains metaplasia in pancreatitis

**DOI:** 10.1096/fj.202201537R

**Published:** 2022-12-05

**Authors:** Baylee J. O'Brien, Erika Y. Faraoni, Lincoln N. Strickland, Zhibo Ma, Victoria Mota, Samantha Mota, Xuebo Chen, Tingting Mills, Holger K. Eltzschig, Kathleen E. DelGiorno, Jennifer M. Bailey‐Lundberg

**Affiliations:** ^1^ Center for Perioperative Medicine, Department of Anesthesiology, McGovern Medical School The University of Texas Health Science Center at Houston Houston Texas USA; ^2^ Gene Expression Laboratory The Salk Institute for Biological Sciences San Diego California USA; ^3^ The Graduate School of Biomedical Sciences The University of Texas MD Anderson Cancer Center and The University of Texas Health Science Center at Houston Houston Texas USA; ^4^ Department of Biochemistry, McGovern Medical School The University of Texas Health Science Center at Houston Houston Texas USA; ^5^ Department of Cell and Developmental Biology Vanderbilt University Nashville Tennessee USA

**Keywords:** acinar‐to‐ductal metaplasia, CD73, inflammation, purinergic signaling

## Abstract

Pancreatitis is currently the leading cause of gastrointestinal hospitalizations in the US. This condition occurs in response to abdominal injury, gallstones, chronic alcohol consumption or, less frequently, the cause remains idiopathic. CD73 is a cell surface ecto‐5′‐nucleotidase that generates extracellular adenosine, which can contribute to resolution of inflammation by binding adenosine receptors on infiltrating immune cells. We hypothesized genetic deletion of CD73 would result in more severe pancreatitis due to decreased generation of extracellular adenosine. CD73 knockout (*CD73* ^−/−^) and C57BL/6 (wild type, WT) mice were used to evaluate the progression and response of caerulein‐induced acute and chronic pancreatitis. In response to caerulein‐mediated chronic or acute pancreatitis, WT mice display resolution of pancreatitis at earlier timepoints than *CD73* ^−/−^ mice. Using immunohistochemistry and analysis of single‐cell RNA‐seq (scRNA‐seq) data, we determined CD73 localization in chronic pancreatitis is primarily observed in mucin/ductal cell populations and immune cells. In murine pancreata challenged with caerulein to induce acute pancreatitis, we compared *CD73* ^−/−^ to WT mice and observed a significant infiltration of Ly6G+, MPO+, and Granzyme B+ cells in *CD73* ^−/−^ compared to WT pancreata and we quantified a significant increase in acinar‐to‐ductal metaplasia demonstrating sustained metaplasia and inflammation in *CD73* ^−/−^ mice. Using neutrophil depletion in *CD73* ^−/−^ mice, we show neutrophil depletion significantly reduces metaplasia defined by CK19+ cells per field and significantly reduces acute pancreatitis. These data identify CD73 enhancers as a potential therapeutic strategy for patients with acute and chronic pancreatitis as adenosine generation and activation of adenosine receptors is critical to resolve persistent inflammation in the pancreas.

**Abbreviations**
ADM: acinar‐to‐ductal metaplasia
AMP: adenosine monophosphate
ATP: adenosine triphosphate
CCK: cholecystokinin
CD39: ectonucleotidase triphosphate diphosphohydrolase‐1
EEC: enteroendocrine
HPLC: high‐performance liquid chromatography
IHC: immunohistochemistry
TNF: tumor necrosis factor α
NECA: 5′‐N‐Ethylcarboxamidoadenosine

## 1 INTRODUCTION

Pancreatitis is an inflammatory condition of the pancreas characterized by severe abdominal pain and the prevalence of acute and chronic pancreatitis is increasing worldwide.^1^ The incidence and morbidity of pancreatitis are increased significantly over the past few decades and acute pancreatitis is now the leading cause of gastrointestinal diagnosis for inpatient hospitalizations in the United States.^1^, ^2^ This condition occurs in response to chronic alcohol consumption, gallstones, abdominal injury, or, less frequently, the cause remains idiopathic.^3^ In alcohol‐associated pancreatitis, alcohol induces the aberrant intracellular activation of trypsin within the pancreatic acini resulting in autodigestion of the organ.^4^ Additionally, alcohol releases the secretagogue cholecystokinin (CCK) from duodenal cells that stimulates secretion of zymogen granules in pancreatic acinar cells resulting in a profound systemic inflammatory response, acinar‐to‐ductal metaplasia (ADM), and fibrosis.^4^ Animal models of acute and chronic pancreatitis have been developed to study mechanisms of acinar cell responses to injury by administering supraphysiological concentrations of caerulein, a CCK analog.^2^

Adenosine (ADO) has been recognized for decades as an important physiologic and pharmacologic regulator that signals through cell surface receptors to regulate cellular functions.^5^, ^6^ More recently, adenosine has been described as paracrine regulator of the tumor microenvironment (TME) and immunosuppressive metabolite elevated in a number of solid tumors including pancreatic ductal adenocarcinoma (PDAC).^7^, ^8^, ^9^, ^10^, ^11^, ^12^, ^13^, ^14^, ^15^, ^16^, ^17^ While inhibitors targeting CD73 or adenosine receptors are therapeutic targets for PDAC patients,^18^ under acute or chronic inflammatory conditions, adenosine can promote fibrosis or reduce inflammation, both critical components of wound healing and repair necessary for tissue regeneration.^19^, ^20^, ^21^, ^22^, ^23^, ^24^, ^25^, ^26^, ^27^, ^28^ Adenosine triphosphate (ATP) is the primary source of energy for cellular processes localized in the intracellular space; however, in response to inflammation or hypoxia, ATP is released into the extracellular space and has been shown to promote pancreatitis.^29^, ^30^ In the presence of ectonucleotidase triphosphate diphosphohydrolase‐1, NTPDase1, (CD39), extracellular ATP is rapidly converted to adenosine diphosphate (ADP) and monophosphate (AMP), which is subsequently converted to adenosine by a cell surface ecto‐5′‐nucleotidase, CD73.^31^, ^32^, ^33^ Generation of extracellular adenosine by CD73 and stimulation of adenosine receptors are shown to promote fibrosis by signaling through A_2A_ and A_2B_ receptors in the lung, skin, and liver. In contrast, acute stimulation of the A_2B_ receptor restrains fibrosis in the heart through reduced fibroblast proliferation and decreased collagen synthesis; however chronic A_2B_ stimulation may promote cardiac fibrosis.^34^, ^35^, ^36^ CD73‐generated adenosine can promote resolution of inflammation by inhibiting the inflammatory function of neutrophils by signaling at higher concentrations via A_1_ or A_2_ receptors to prevent tissue injury from prolonged inflammatory responses.^27^, ^32^, ^37^, ^38^, ^39^, ^40^ In models of sepsis, agents with nucleoside triphosphate hydrolase activity targeting CD39 reduced platelet–leukocyte–endothelium interactions, reduced pro‐inflammatory cytokines, and prolonged survival.^41^ The adenosine ENT1 transporter facilitates the uptake of extracellular adenosine across the cell membrane and is one mechanism to downregulate extracellular concentrations of adenosine.^42^ Repressing ENTs in the lung has been shown to reduce inflammatory lung injury.^43^ Adenosine signals via G‐protein coupled receptors: A_1_, A_2A_, A_2B_, and A_3_.^44^, ^45^, ^46^, ^47^ The A_1_ G_i_‐coupled receptor has a high affinity for adenosine, while the A_2A_ and A_2B_ G_s_‐coupled receptors have a lower affinity. Additionally, A_3_ receptor affinity for adenosine was shown to rank higher than A_2B_; however, discrepancies in the role of A_3_ receptors have also been observed in the literature, presenting both anti‐inflammatory and pro‐inflammatory effects.^44^, ^48^, ^49^, ^50^, ^51^, ^52^, ^53^, ^54^ Therefore, at early stages of inflammation, low local concentrations of adenosine may promote immune recruitment via the A_1_ receptor while later high adenosine concentrations can suppress immune activity via the A_2A_, A_2B_, or A_3_ receptors.^44^, ^52^, ^55^

Adenosine binding to adenosine receptors modulates both the innate and the adaptive immune response to hypoxia, inflammation, and tissue repair.^33^ The A_2A_ and A_2B_ receptors exert anti‐inflammatory effects by inhibiting neutrophil chemotaxis, attachment to vascular endothelial cells, and phagocytosis.^14^, ^44^, ^56^, ^57^ In the pancreas, A_2A_ has been shown to protect against pancreatic dysfunction, islet size, and insulin context in the context of high‐fat diet‐induced diabetes and obesity.^58^ Inflammatory macrophages are inhibited through A_2A_ and A_2B_ activation resulting in decreased production of cytokines including IL‐1β, IL‐18, IL‐6, and TNF‐α.^14^, ^32^ Additionally, CD4+ T cell activation and proliferation and natural killer cell cytotoxic functions are inhibited by A_2A_ receptor activation.^14^ Additionally, A_3_ enhancement was reported to inhibit macrophage inflammatory protein (MIP)‐1α in a model of collagen‐induced arthritis,^53^ and two other murine studies of colitis showed reduced inflammation and increased survival following A_3_ activation.^54^

In contrast, decreased activity of CD73 and extracellular adenosine is associated with amplified activation and chemotactic functions of immune cell populations.^32^ The purinergic P2X and P2Y family of receptors are expressed on neutrophils and promote neutrophil‐mediated oxidative burst‐induced tissue injury in the presence of ATP.^59^

In response to injury, pancreatic acini can undergo acinar‐to‐ductal metaplasia (ADM), a metaplastic event that limits pancreatic tissue damage via a rapid decline in zymogen production.^60^, ^61^, ^62^ Experimental studies have shown injured acinar cells activate a shift in gene expression regulated by Mist1 and Ptf1α to transdifferentiate away from their specified cell type and function, which consists of highly specialized cells involved in the production and secretion of digestive enzymes, toward a ductal phenotype.^57^, ^63^ ADM trans‐differentiation is also triggered by innate and pro‐inflammatory immune cells, including neutrophils and macrophages, that infiltrate the pancreas resulting in elevated secretion of inflammatory cytokines including RANTES and tumor necrosis factor α (TNF).^64^ Differentiation into a cell type with ductal characteristics demonstrates a pancreatic repair process under strong positive selection in pancreatitis.^23^, ^26^ These mucinous populations can subsequently seed tuft cell and enteroendocrine lineages as further reparative mechanisms.^65^

## 2 MATERIALS AND METHODS

### 2.1 Animal model

All mouse model procedures are in compliance with UTHealth's CLAMC Animal Welfare Committee Review and approved on Dr. Bailey‐Lundberg's AWC protocol. To evaluate the role of CD73 in pancreatitis, CD73 knockout (*CD73* ^−/−^) and C57BL/6 (wild type) mice were used. Full‐body CD73KO mice were purchased from The Jackson Laboratory strain 018986. Controls for each experiment were derived from wild‐type crosses. Caerulein injections were performed for each experiment during Spring months (March–June) 2022 and mixed genders were equally included for all groups. In the acute pancreatitis model, *CD73* ^−/−^ and wild‐type mice were intraperitoneally injected on alternating flanks with 70 μg/kg of caerulein (Sigma Aldrich 17650‐98‐5) 8× a day for two consecutive days. Mice were euthanized at 1, 4, and 7 days after the last caerulein injection to evaluate ADM abundance, inflammatory progression, and organ repair. For the chronic model, *CD73* ^−/−^ and wild‐type mice were injected intraperitoneally on alternating flanks with 250 μg/kg of caerulein (Sigma Aldrich 17650‐98‐5) twice a day, 5 days a week, for 2 weeks. The mice were allowed to recover for 2 days after the last injection before euthanasia by isoflurane overdose.

### 2.2 Neutrophil depletion in vivo

*CD73* ^−/−^ mice were intraperitoneally administered 300 μg of anti‐Ly6G antibody (clone1A8, BioXCell, West Lebanon, NH) at Day ‐2, 0, and 1 day after the last caerulein injection. IgG2a isotype (clone 2A3, BioXCell, West Lebanon, NH) was used for control. Mice were euthanized at Day 4.

### 2.3 NECA in vivo

*CD73* ^−/−^ and wild‐type mice under acute pancreatitis protocol of eight injections per day during two consecutive days (Days ‐1 and 0) were administered a single injection of 80 μg/kg of 5′‐N‐Ethylcarboxamidoadenosine (NECA) (MedChemExpress HY‐103173) after the sixth injection of caerulein on day zero. Mice were euthanized at Day 1. NECA injection was performed intraperitoneally.

### 2.4 Immunohistochemistry and ImageJ analysis

Tissues were fixed in zinc‐buffered formalin, processed according to standard protocols, and embedded in paraffin. The unstained sections were baked at 60°C for 45 min. The sections were deparaffinized with Histoclear and rehydrated stepwise. Heat‐mediated antigen retrieval was followed with a pH 6 unmasking solution (Vector Laboratories, H‐3300) and a pH 9 unmasking solution (Abcam 100X Tris‐EDTA). All sections were blocked for 1 h in 10% FBS in PBST. Primary antibodies were used at a 1:200 dilution and incubated overnight at 4°C. Secondary antibodies were used at a 1:500 dilution and incubated at room temperature for 30 min. The Vectastain ABC kit Peroxidase Standard (Vector Laboratories, PK4000) and DAB Peroxidase (HRP) Substrate kit (Vector Laboratories, SK‐4100) were used. Human pancreatitis tissues were obtained from a TMA array (US Biomax, Inc. BIC14011b), where all tissues were fixed in 10% neutral formalin for 24–48 h, dehydrated with gradient ethanol, cleared with xylene, and embedded in paraffin. All human tissues from US Biomax TMA BIC14011b were collected under HIPPA‐approved protocols and approved for commercial product development. Six independent chronic pancreatitis cores were analyzed for CD73 expression (Abcam, cat. Ab133582) following the IHC protocol mentioned above.

Image analysis was performed using ImageJ (http://imagej.nih.gov/ij/) software and three to five representative fields per tissue were used depending on the size of the tissue. The color threshold tool was used to determine positive staining and ensure normalization of all samples. Freehold selection tool was used to isolate mild and severe pancreatitis from normal tissue. Pancreatitis areas were defined by the presence of swelling between pancreatic lobes, significant infiltrating immune cells, ADM cells, and loss of normal cellular histology.

### 2.5 Nucleoside purification

Pancreas tissue was collected at the end of the experiment and flash frozen. At time of experiment, tissue was homogenized in perchloric acid using a Beadbug microtube homogenizer (BeadBug™, cat. SKUD1036). Pancreas homogenates were then centrifuged at 14 000 rpm at 4°C for 10 min and the supernatant was collected. A Pierce™ BCA Protein Assay Kit (Thermo Scientific, cat. 23225) was performed to determine protein concentration following manufacturer instructions. Samples were then neutralized with phenol red and KHCO3/KOH, vortexed, acidified with (NH_4_)_3_PO_4_ and H_3_PO_4_, then vortexed. Samples were centrifuged at 14 000 rpm for 5 min and 1 ml of supernatant was collected and filtered for further analysis.

### 2.6 High‐performance liquid chromatography (HPLC)

Filtered supernatants were analyzed by high‐performance liquid chromatography using the Waters Breeze 2 HPLC System (Waters 2489 UV/Visible Detector and Waters 1525 Binary HPLC Pump). Flow rate was 1 ml/min and 100 μl per sample was injected in the column (XSElect HSS C18 SB 5 μm 4.6 × 250 mm) with a mobile phase 100% A (0.02M NH_4_H_2_PO_4_) for 0–4 min, which then was switched to 100% B (0.02M NH_4_H_2_PO_4_ containing 20% methanol) from 4 to 8 min, then stayed in 100% B from 8 to 18 min, and finally switched back to 100% A from 18 to 20 min. Absorbance was measured at a wavelength of 260 and 280 nm, and adenosine and AMP peaks were determined using a standard HPLC curve. Pancreas tissue adenosine and AMP levels were normalized to lysate protein levels.

### 2.7 Analysis of published single‐cell RNA sequencing datasets

Processed count matrices for scRNA‐seq datasets from Ma et al. were downloaded from the Gene Expression Omnibus (GEO) database (accession number GSE172380). The processed human pancreas sNuc‐seq dataset from Tosti et al. was obtained from http://singlecell.charite.de/pancreas/.^65^, ^66^ Low‐quality cells were filtered on read counts, the number of genes expressed, and the ratio of mitochondrial reads following the thresholds described in the respective publications. Filtered gene count matrices were log‐normalized, and the top 2000 variable features were further scaled prior to dimension reduction by PCA and being embedded in UMAP using the R package Seurat.^67^ Seurat cell clusters were labeled with major cell types using marker genes provided by the authors.

## 3 RESULTS

### 3.1 CD73 is expressed on ductal cells in human chronic pancreatitis

**FIGURE 1:**
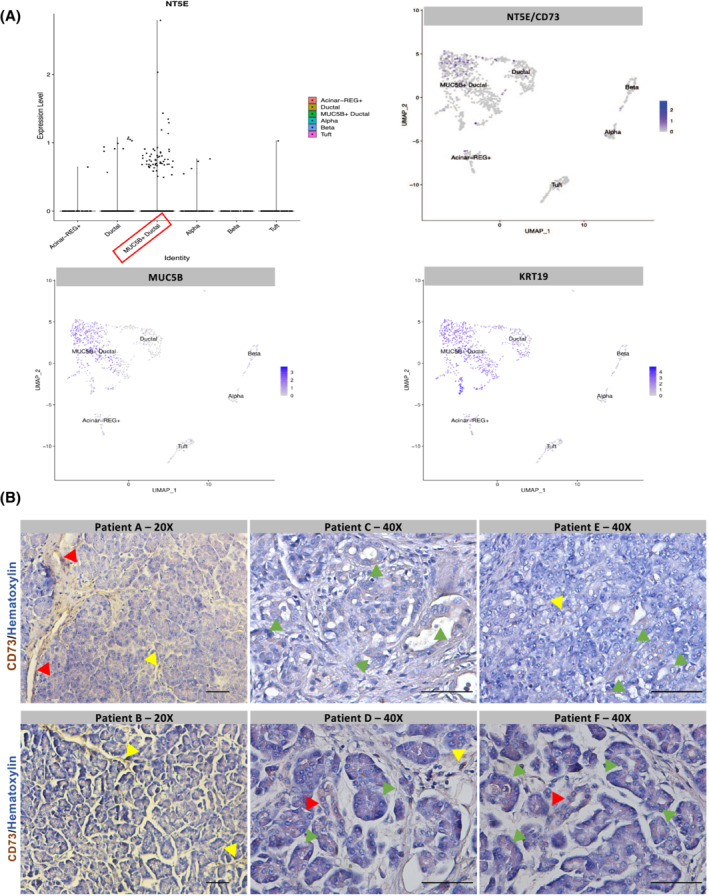
CD73 is expressed on ductal cells and infiltrating immune cells in human chronic pancreatitis. Single‐nuclear RNA sequencing of human chronic pancreatitis was analyzed. IHC for CD73 was performed in a TMA with six human cases of chronic pancreatitis. (A) Single‐nuclear RNA sequencing^65^ and associated UMAP from human chronic pancreatitis. NT5E (CD73) is expressed in a MUC5B+ ductal cell population (MUC5B+ and KRT19+ cells). (B) Human chronic pancreatitis tissue (*n* = 6) demonstrating positive CD73 staining in ADM cells (green arrows), ductal cells (red arrows), and infiltrating immune cells (yellow arrows). Bars represent 50 μM.

Recent literature has described that CD73 is elevated in PDAC epithelial cells resulting in adenosine generation and an immune‐suppressive tumor microenvironment.^9^, ^10^, ^11^ In the normal pancreas, CD73 is expressed in vascular cells, but limited to no expression is observed in acinar or ductal cells. In contrast, NTPdase1 (CD39) is expressed in acinar cells and blood vessels, which have ATPase activity; yet CD39 is expressed at low levels in normal pancreatic ducts.^68^, ^69^ As chronic pancreatitis is a risk factor for development of PDAC,^70^ and divergent roles for adenosine have been described in inflammatory diseases, we wanted to investigate the cell‐type‐specific localization of CD73 in human and murine chronic pancreatitis and determine if CD73 is an important determinant of pancreatitis severity. To evaluate cellularity of CD73 expression, we analyzed previously published single‐nucleus RNA‐sequencing data (sNuc‐seq) generated from two patients with chronic pancreatitis (totaling 2726 nuclei).^65^ These data revealed *NT5E*, the gene encoding for CD73, is highly expressed in a MUC5B+ ductal cell population (Figure 1A). To confirm ductal cells expressed CD73, we used a human tissue microarray to evaluate cellularity of CD73 immunolabeling in chronic pancreatitis. An immunohistochemistry (IHC) stain for CD73 in 6 patient samples from human chronic pancreatitis (Figure 1B) revealed a strong positive staining in ADM cells (green arrows), ductal cells (red arrows), and infiltrating immune cells (yellow arrows).

### 3.2 Genetic loss CD73 increases severity of chronic pancreatitis

**FIGURE 2:**
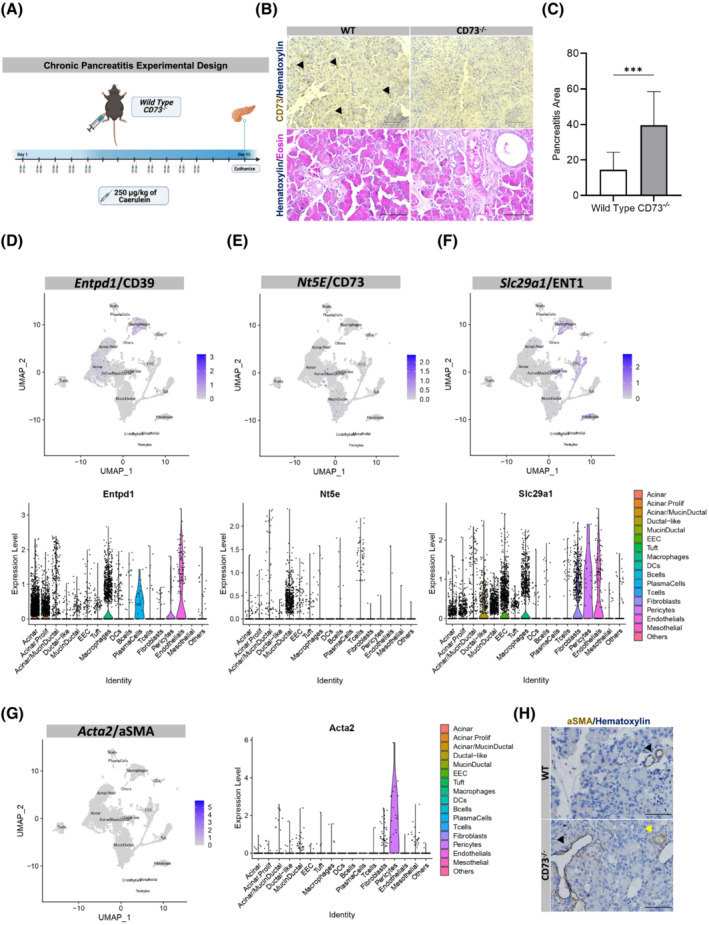
Genetic loss of CD73 increases severity of chronic pancreatitis. An in vivo model of chronic pancreatitis was performed in WT and *CD73* ^−/−^ mice. Histopathology and purinergic enzymes and stellate cell marker expression were analyzed. (A) Experimental design for the chronic pancreatitis model in vivo. (B) IHC stain for CD73 in WT and *CD73* ^*−/−*^ mice demonstrating positive staining on infiltrating immune cells as well as ductal cells only in WT mice (Top panel, black arrows). H&E stain of chronic pancreatitis in WT and *CD73* ^*−/−*^ mice (Bottom panel). (C) ImageJ quantification of severe chronic pancreatitis revealed significantly increased severe pancreatitis area *CD73* ^*−/−*^ mice compared to WT. Data were analyzed by Student's *t* test. (D–F) Single‐cell RNA sequencing and associated UMAP from a chronic pancreatitis mouse model at 2 and 4 weeks^65^ showed *Entpd1*/CD39 is highly expressed in acinar cells and macrophages (D), *Nt5e*/CD73 is highly expressed in mucin/ductal cell populations (E), *Slc29a1*/ENT1 adenosine transporter is highly expressed in ductal‐like, endothelial, EEC cell populations and macrophages (F) and (G) *Acta2*/aSMA is expressed mainly by pericytes, mucin‐ductal, and fibroblast cells. (H) IHC showing detectable aSMA expression in small vessels (black arrows) and duct‐like structures (yellow arrows) but undetected in fibroblast cells in both WT and *CD73* ^*−/−*^ mice. Scale bars 500 μm. Error bars, SEM. ***p ≤ .001.

To determine the role of CD73 in pancreatitis, we utilized a murine 2‐week chronic pancreatitis model (Figure 2A). Wild type and *CD73* ^−/−^ mice were used for the study, and both genotypes were subjected to a caerulein‐induced pancreatitis protocol consisting of two 250 μg/kg injections per day, 5 consecutive days a week, for 2 weeks. The mice were then euthanized after a 2‐day recovery period to determine expression of CD73 in murine pancreatitis and to evaluate if loss of CD73 resulted in any histopathologic changes in the pancreas compared to wild‐type pancreata. We determined the localization of CD73 in vivo by CD73 IHC staining and observed a strong positive expression of CD73 on infiltrating immune cells as well as ductal cells only in wild‐type mice under chronic pancreatitis, whereas the absence of CD73 expression was confirmed in pancreata from the in *CD73* ^−/−^ mice (Figure 2B, Top panels, black arrows).

To evaluate tissue injury and pathology in the context of chronic caerulein treatment, we used Hematoxylin & Eosin (H&E) to stain wild‐type and *CD73* ^−/−^ mice (Figure 2B, Bottom panels). For comparison, severe pancreatitis was quantified and defined as the presence of significant infiltrating immune cells, ADM cells, and loss of normal cellular histology. Under caerulein‐mediated chronic pancreatitis conditions, *CD73* ^−/−^ mice displayed significantly increased severe pancreatitis area per field (*p* < .001) (Figure 2C) suggesting the loss of extracellular adenosine generation exacerbates and sustains tissue injury as well as inhibits tissue regeneration.

As we observed such a prominent difference in pancreatic injury in wild type compared to *CD73* ^−/−^ mice after chronic injection of caerulein, we wanted to evaluate the cellular expression of CD73, CD39, and ENT1 in caerulein‐mediated murine chronic pancreatitis. We analyzed single‐cell RNA sequencing data from a chronic caerulein‐mediated mouse model recently published by Ma et al. encompassing ~21 140 cells from four mice.^65^ The results demonstrated the enzyme CD39, responsible for catalyzing the conversion of ATP to ADP and AMP, is highly expressed on macrophages, pericytes, endothelial cells, and acinar cells (Figure 2D); whereas, similar to what we identified in human chronic pancreatitis, CD73 is highly expressed in a mucin/ductal cell population as well as in T cells, macrophages, and B cells (Figure 2E). Additionally, we observed the ENT1 adenosine transporter that facilitates the movement of extracellular adenosine across the cell membrane is highly expressed in macrophages, fibroblasts, pericytes, enteroendocrine cells, endothelial cells, and ductal‐like cells (Figure 2F). Lastly, given its role in chronic pancreatitis and fibrotic development,^71^ the expression of aSMA, a common marker of activated stellate cells, was studied. Single‐cell RNA sequencing analysis showed *Acta2*, the gene encoding for aSMA, expression mainly in pericytes, mucin‐ductal, and fibroblast cells during chronic pancreatitis (Figure 2G); however, when analyzed by IHC (Figure 2H) in both WT and *CD73* ^−/−^ mice, aSMA protein expression was not detected in fibroblasts; however, aSMA expression was clearly observed in small vessels (black arrows) and mucin‐ductal structures (yellow arrow), suggesting little or undetected activation of stellate cells in this experimental mouse model of chronic pancreatitis by the above‐mentioned technique.

### 3.3 Purinergic signaling modulates response to acute pancreatitis

**FIGURE 3:**
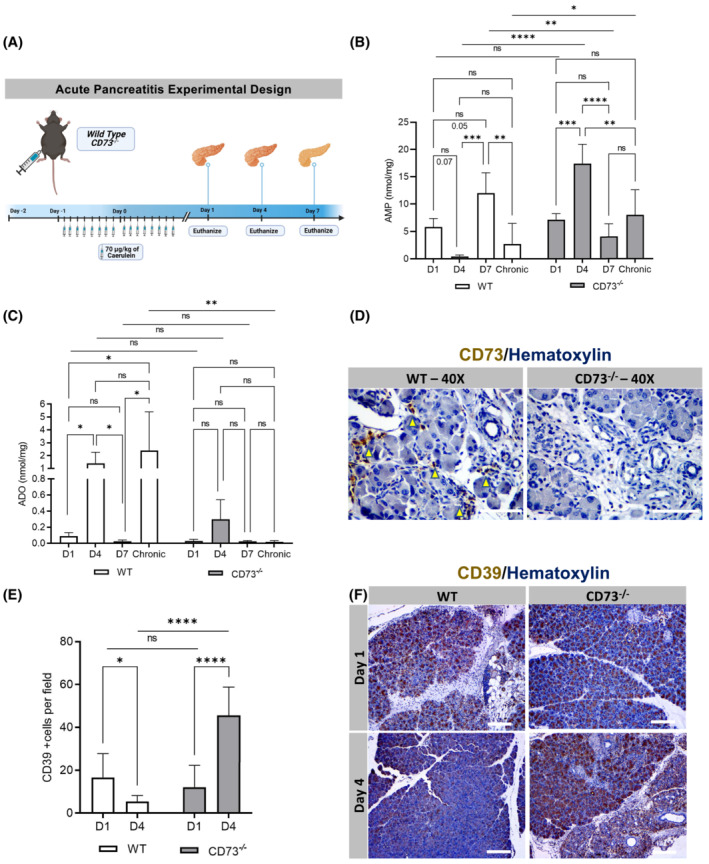
Purinergic signaling modulates response to acute pancreatitis. AMP and ADO levels were analyzed by HPLC from pancreatic tissues for AMP in WT mice at Days 1 (*n* = 4), 4 (*n* = 4), 7 (*n* = 3) and chronic model (*n* = 5) and *CD73* ^*−/−*^ mice at Days 1 (*n* = 5), 4 (*n* = 4), 7 (*n* = 4), and chronic model (*n* = 5). CD39 and CD73 expressions were analyzed by IHC. (A) Experimental design for the acute pancreatitis model in vivo. (B) AMP levels acutely decrease from Day 1 to Day 4 and increase from Day 4 to Day 7 in WT mice; in contrast, in *CD73* ^−/−^ mice these levels increase from Day 1 to Day 4 and decrease from Day 4 to Day 7. Chronic exposure to caerulein showed increased AMP levels in *CD73* ^*−/−*^ mice. (C) ADO levels increased in WT mice between Day 1 and Day 4, followed by a decrease between Day 4 and Day 7; however, no significant variations were observed in *CD73* ^*−/−*^ mice. Chronic exposure to caerulein showed decreased ADO levels when compared with WT group. D1 and D4 were compared with Student's *t* test. (D) CD73 at Day 4 revealed positive staining on infiltrating immune cells (yellow arrows) in the WT only. (E) ImageJ quantification of CD39 showed decreased expression between Day 1 and Day 4 in WT mice and increased in *CD73* ^*−/−*^ murine pancreata. Additionally, at Day 4 a significant increase in CD39 expression was observed in *CD73* ^*−/−*^ compared to the WT pancreata. (F) IHC stain for CD39 in WT and *CD73* ^*−/−*^ mice at Day 1 and 4. Data were analyzed by two‐way ANOVA. Error bars, SEM. *p ≤ .05; **p ≤ .01; ***p ≤ .001; ****p ≤ .0001; n.s., not significant. Scale bars 50 μm.

As we observed such a significant difference in pancreatitis area in the chronic pancreatitis model, we wanted to evaluate the role of CD73 in a caerulein‐mediated acute pancreatitis model, which allows for histologic visualization of pancreatic repair over a time frame of 7 days after acute injury.^72^ Wild type and *CD73* ^−/−^ mice were used for the study and underwent a caerulein‐induced acute pancreatitis protocol which consisted of eight injections of 70 μg/kg caerulein per day for two consecutive days (Figure 3A). Mice were then euthanized at 1, 4, and 7 days after the last caerulein injection to evaluate the timing of AMP and adenosine generation, which were also compared to chronic exposure to caerulein levels. Under acute pancreatitis, high‐performance liquid chromatography (HPLC) revealed AMP levels acutely decrease from Day 1 to Day 4, and increase from Day 4 to Day 7 in wild‐type mice; in contrast, in *CD73* ^−/−^ mice these levels increase from Day 1 to Day 4 and decrease from Day 4 to Day 7, suggesting a transient accumulation of AMP in the context of no CD73 activity (Figure 3B). In wild‐type mice, acute pancreatitis showed AMP variations that were accompanied by a significant increase in ADO levels between Day 1 and Day 4, followed by a decrease between Day 4 and Day 7; however, no significant variations were observed in *CD73* ^−/−^ mice (Figure 3C). Chronic exposure to caerulein showed increased AMP levels in *CD73* ^−/−^ mice but decreased ADO levels when compared with wild‐type pancreata.

ADO levels were significantly elevated at Day 4 during acute pancreatitis so we wanted to determine the localization of CD73 at Day 4. IHC showed CD73 expression in infiltrating immune cells as well as ductal cells (Figure 3D, yellow arrows) in wild‐type mice. CD73 staining was also performed on *CD73* ^−/−^ mice to confirm the genotype, which correctly demonstrated negative staining of the tissue.

To compare the initial modulation in nucleotide generation under acute conditions in wild‐type and *CD73* ^−/−^ mice, an IHC antibody stain for CD39 was performed at Day 1 and Day 4. CD39 expression decreased between Day 1 and Day 4 in wild‐type mice and increased in *CD73* ^−/−^ murine pancreata (Figure 3E,F). Additionally, at Day 4 a significant increase in CD39 expression was observed in *CD73* ^−/−^ compared to the wild‐type pancreata. These data support the HPLC data that *CD73* ^−/−^ mice are experiencing enhanced nucleotide or purinergic signaling at Day 4, which may be a major determinant of sustained tissue injury in *CD73* ^−/−^ mice, while the wild‐type mice utilize CD73 to convert AMP to adenosine.

### 3.4 Genetic loss of CD73 promotes metaplasia in acute pancreatitis

**FIGURE 4:**
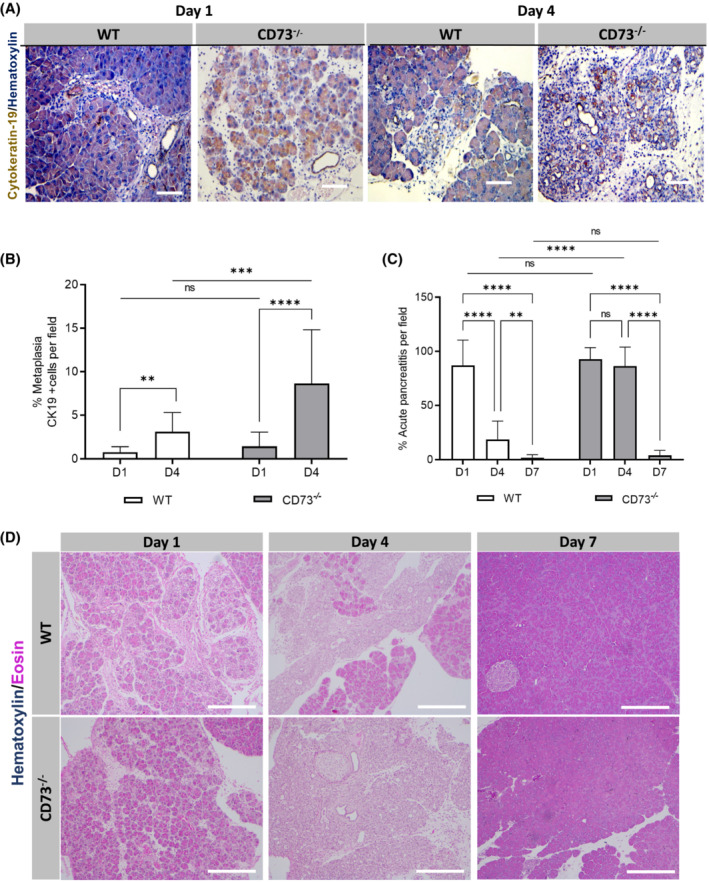
Genetic loss of CD73 promotes metaplasia in acute pancreatitis. Metaplasia was studied by Cytokeratin 19 IHC staining in WT and *CD73* ^*−/−*^ mice at Days 1 and 4. Pancreatitis area was evaluated in WT mice at Days 1 (*n* = 12) and 4 (*n* = 14) and in *CD73* ^*−/−*^ mice at Days 1 (*n* = 12) and 4 (*n* = 21). (A) IHC stain for Cytokeratin‐19 in WT and *CD73* ^*−/−*^ mice at Days 1 and 4. (B) ImageJ quantification of Cytokeratin‐19‐positive areas per field a significant increase from Day 1 to Day 4 in both genotypes. Additionally, at Day 4, the presence of Cytokeratin‐19+ cells was significantly increased in *CD73* ^*−/−*^ mice compared to WT. Student's *t* test was used to compare WT timepoints. (C) ImageJ quantification of acute pancreatitis revealed increased persistent pancreatitis in *CD73* ^*−/−*^ mice compared to WT. By Day 7, both genotypes demonstrated similar histology with no significant difference in pancreatitis. (D) H&E stain of acute pancreatitis in wild type and *CD73* ^*−/−*^ mice at Days 1, 4, and 7. Data were analyzed by two‐way ANOVA. Error bars, SEM. **p ≤ .01; ***p ≤ .001; ****p ≤ .0001; n.s., not significant. Scale bars 50 μm.

In order to better comprehend the initial tissue injury response and resolution in the acute pancreatitis model, we evaluated immune infiltration and metaplasia. To correlate tissue injury to the amount of ADM cells present, an IHC antibody stain for Cytokeratin‐19, a marker for ductal cells, was performed in wild‐type and *CD73* ^−/−^ mice at Day 1 and Day 4 (Figure 4A). At Day 1, the number of ductal cells per field between experimental groups was similar, indicating comparable initial tissue injury and metaplasia (*p* = n.s.) (Figure 4B). However, at Day 4, *CD73* ^−/−^ mice displayed a significant increase in the amount of Cytokeratin‐19+ areas per field, indicating a significant increase in metaplastic ducts and ADM, compared to wild‐type pancreata (*p* < .001). Interestingly, from Day 1 to Day 4 there was a significant increase in Cytokeratin‐19+ cells in both wild‐type mice (*p* < .01) and *CD73* ^−/−^ mice (*p* < .0001) (Figure 4B). These findings indicate the ADM process is a reparative mechanism concurrent with peak pancreatic adenosine generation.

To evaluate severity of pancreatitis at Day 4 and Day 7, an H&E stain was performed in wild type and *CD73* ^−/−^ mice at Day 1, 4, and 7. At Day 1, both experimental groups showed increased fluid between the pancreatic lobes and acinar cells, a similar presence of ADM cells, and infiltrating immune cells (Figure 4C,D). At Day 4, wild‐type mice demonstrated a return to normal histology characterized by the disappearance of ADM cells and infiltrating immune cells and reduction in excess fluid, while *CD73* ^−/−^ mice showed significantly increased residual pancreatitis areas (*p* < .0001). Lastly, at Day 7 both experimental groups demonstrated near‐complete return to normal histology with no significant difference between them (*p* = n.s.). These data suggest *CD73* ^−/−^ mice exhibit sustained tissue injury and require more time for tissue regeneration compared to the wild‐type mice.

### 3.5 Loss of adenosine increases immune infiltration in acute pancreatitis

**FIGURE 5:**
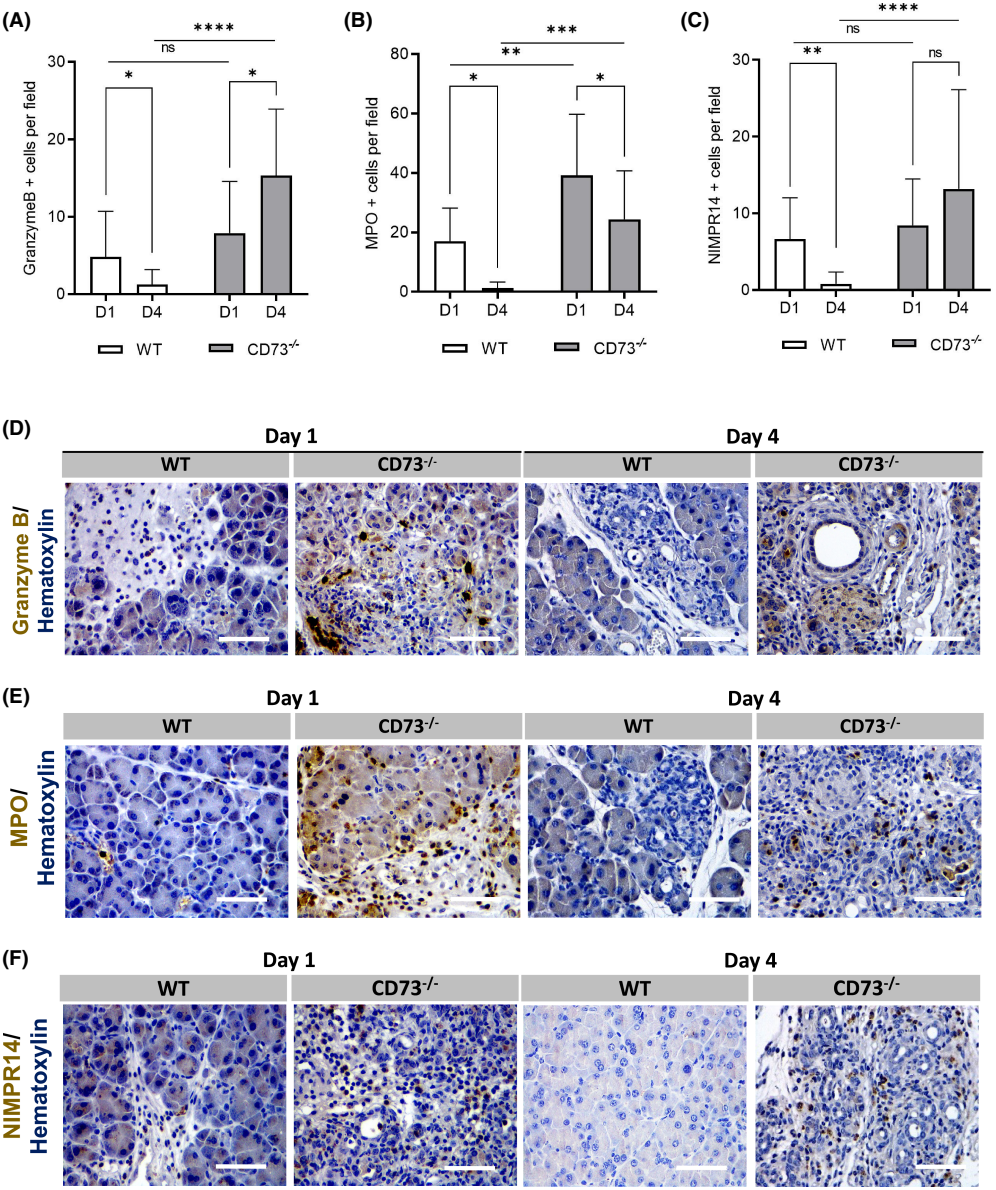
Loss of adenosine increases immune infiltration in acute pancreatitis. Immune cell infiltration was analyzed by IHC in WT and *CD73* ^*−/−*^ mice at days 1 and 4. (A) ImageJ quantification of Granzyme B+ cells per field, a marker for T‐cells and Natural Killer cells, was increased only in *CD73* ^*−/−*^ mice at D4 when compared with D1 and WT D4 measurements. WT mice presented decreased Granzyme B+ cells at D4 compared to D1. Student's *t* test was used to analyze WT timepoints. (B) ImageJ quantification of MPO+ cells per field showed a decrease from D1 to D4 in both genotypes. Additionally, *CD73* ^*−/−*^ mice presented increased MPO‐positive cells at both timepoints when compared with WT mice. (C) ImageJ quantification of NIMPR‐14+ cells showed significantly increased staining in CD73^−/−^ mice when compared with WT. In WT mice, levels decreased from D1 to D4. Student's *t* test was used to analyze WT timepoints. Representative images for Granzyme B (D), MPO (E), and NIMPR14 (F) IHC staining. Data were analyzed by two‐way ANOVA. Error bars, SEM. *p ≤ .05; **p ≤ .01; ***p ≤ .001; ****p ≤ .0001; n.s., not significant. Scale bars 50 μm.

To evaluate mediators of sustained inflammation in the *CD73* ^−/−^ mice, we used IHC to stain for Granzyme B, Myeloperoxidase (MPO) and NIMPR14, a neutrophil marker. IHC experiments were performed in wild‐type and *CD73* ^−/−^ mice at Day 1 and Day 4. At Day 1, the number of Granzyme B+ cells was similar between experimental groups (*p* = n.s.) (Figure 5A,D). However, at Day 4 *CD73* ^−/−^ mice demonstrated significantly increased Granzyme B+ cells compared to the wild‐type mice (*p* < .0001). Additionally, from Day 1 to Day 4 there was a significant increase in Granzyme B+ cells in *CD73* ^−/−^ mice (*p* < .05) and a significant decrease in the wild type (*p* < .05). These data suggest Granzyme B expressing cells are a major determinant of pancreatitis severity in the absence of extracellular adenosine.

To determine the innate immune system's role in initial tissue injury and resolution, an IHC stain for MPO, a marker for inflammatory neutrophils, was performed in wild‐type and *CD73* ^−/−^ mice at Day 1 and Day 4 (Figure 5B,E). From Day 1 to Day 4 there was a significant decrease in MPO+ cells in both wild‐type and *CD73* ^−/−^ mice (*p* < .05). Interestingly, *CD73* ^−/−^ mice demonstrated a significantly increased amount of MPO+ cells per field compared to the wild‐type mice at both Day 1 and Day 4 measurements (*p* < .01; *p* < .001, respectively), suggesting the absence of extracellular adenosine in *CD73* ^−/−^ mice promotes early tissue injury via innate immune cell infiltration.

Due to the potent induction of the innate immune system at Day 1 as shown in the MPO IHC, we decided to specifically evaluate neutrophil activity. An IHC stain for NIMPR‐14, a marker for Ly6G+ and Ly6C+ neutrophils, was performed in wild‐type and *CD73* ^−/−^ mice at Day 1 and Day 4 (Figure 5C,F). At Day 1, the number of neutrophils per field was similar between experimental groups (*p* = n.s.); however, at Day 4 *CD73* ^−/−^ mice demonstrated significantly increased neutrophils per field compared to the wild‐type (*p* < .0001). From Day 1 to Day 4 there was a significant decrease in neutrophils in wild‐type mice (*p* < .01); while no difference was observed in neutrophil abundance in the *CD73* ^−/−^ (*p* = n.s.). These results suggest that the sustained tissue injury seen in *CD73* ^−/−^ mice at Day 4 is primarily due to the continued induction and activation of neutrophils in the absence of extracellular adenosine and possibly due to heightened ATP or AMP‐dependent purinergic signaling.

### 3.6 Adenosine restrains neutrophil‐mediated tissue injury

**FIGURE 6:**
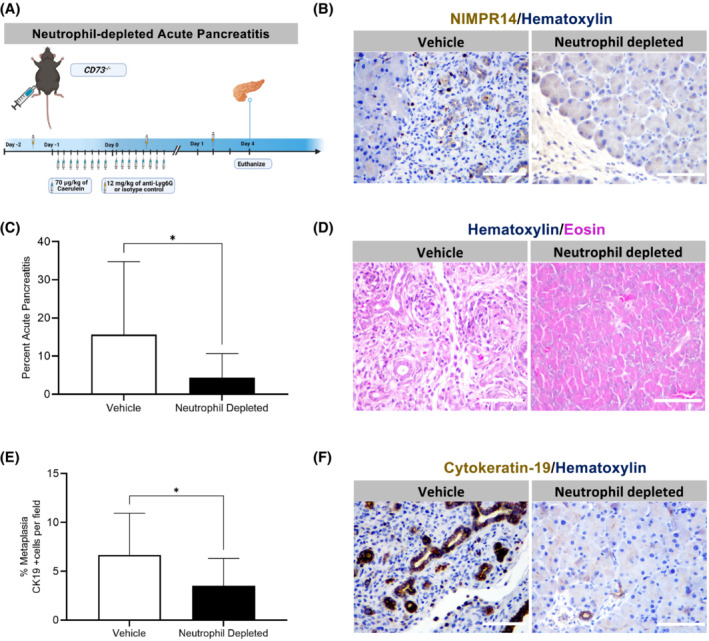
Genetic loss of adenosine generation by CD73 increases neutrophil‐mediated oxidative stress‐induced tissue injury. A neutrophil depletion in vivo experiment was performed in WT and *CD73* ^*−/−*^ mice subjected to acute pancreatitis. Mice were euthanized at Day 4. Pancreatitis and cytokeratin 19 areas were assessed by histopathology and IHC. (A) Experimental design for the neutrophil‐depleted acute pancreatitis model in vivo. (B) IHC stain for NIMPR‐14 was performed in vehicle‐treated and neutrophil‐depleted *CD73* ^*−/−*^ mice at Day 4 to confirm depletion. (C) ImageJ quantification at day 4 revealed decreased pancreatitis area per field in neutrophil‐depleted mice compared to vehicle‐treated. (D) H&E stain in neutrophil‐depleted acute pancreatitis mice model at Day 4. (E) ImageJ quantification of Cytokeratin‐19‐positive areas per showed decreased levels in neutrophil‐depleted mice compared to vehicle‐treated. (F) IHC stain for Cytokeratin‐19 in neutrophil‐depleted acute pancreatitis mice model at Day 4. Data were analyzed by Student's *t* test. Error bars, SEM. *p ≤ .05. Scale bars 50 μm.

To confirm that adenosine generation promoted neutrophil‐mediated tissue injury, we modified the caerulein‐induced acute pancreatitis model by treating *CD73* ^−/−^ mice with a Ly6G neutrophil depletion antibody or vehicle (Figure 6A) and euthanized the mice at Day 4 post last caerulein injection. To confirm neutrophil depletion, an IHC stain for NIMPR‐14 was performed which demonstrated the presence of neutrophils in the vehicle‐treated and the absence of neutrophils in pancreata from the neutrophil‐depleted mice (Figure 6B). We performed an H&E stain to assess tissue injury, and found that at Day 4, neutrophil‐depleted *CD73* ^−/−^ mice demonstrated significantly less pancreatitis per field compared to vehicle‐treated (*p* < .05) (Figure 6C,D), indicating enhanced neutrophil activity from loss of CD73 dependent adenosine generation promotes sustained inflammation.

To determine if neutrophil‐depletion could prevent metaplasia in acute pancreatitis, an IHC stain for Cytokeratin‐19 was performed in vehicle‐treated and neutrophil‐depleted *CD73* ^−/−^ mice at Day 4, which showed vehicle‐treated mice, compared to neutrophil‐depleted mice, present significantly increased amounts of metaplasia per field, suggesting the absence of neutrophils restrains metaplasia in acute pancreatitis (*p* < .05) (Figure 6E,F).

### 3.7 Enhanced adenosine receptor activation reduces caerulein‐induced acute metaplasia

**FIGURE 7:**
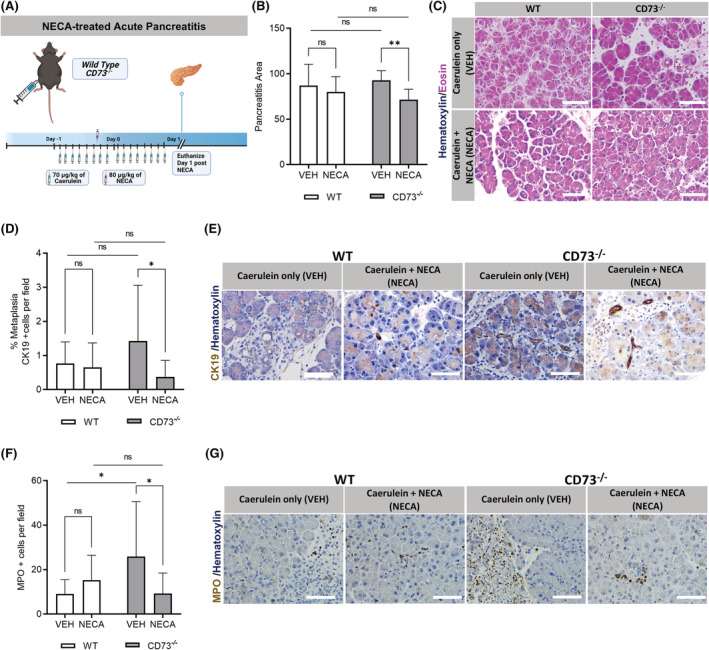
Enhanced adenosine receptor activation reduces inflammation after acute caerulein treatment. An acute pancreatitis in vivo experiment was performed in *CD73* ^*−/−*^ mice treated with NECA or vehicle. Pancreatitis and Cytokeratin 19+ areas were assessed by histopathology and IHC. (A) Experimental design for the NECA‐treated acute pancreatitis model in vivo. (B) ImageJ quantification of pancreatitis per field revealed NECA‐treated *CD73* ^*−/−*^ presented less pancreatitis area than vehicle‐treated mice after acute pancreatitis induction. (C) H&E stain of VEH and NECA‐treated WT and *CD73* ^*−/−*^ mice at Day 1 in the acute pancreatitis model. (D) ImageJ quantification of Cytokeratin‐19‐positive areas per field showed increased metaplastic ductal cells per field in caerulein‐only‐treated *CD73* ^*−/−*^ mice compared to NECA‐treated. (E) IHC stain for Cytokeratin‐19 in VEH‐ and NECA‐treated WT and *CD73* ^*−/−*^ mice at Day 1. (F) ImageJ quantification of MPO‐positive cells per field showed increased levels in cerulean‐only treated *CD73* ^*−/−*^ mice compared to WT mice. Additionally, NECA treatment significantly reduced MPO‐positive cells only in *CD73* ^*−/−*^ mice. (G) IHC stain for MPO in VEH and NECA‐treated WT and *CD73* ^*−/−*^ mice at Day 1. Data were analyzed by two‐way ANOVA. Error bars, SEM. *p ≤ .05; **p ≤ .01; n.s., not significant. Scale bars 50 μm.

To determine if enhanced adenosine receptor activation would reduce tissue injury and metaplasia, wild‐type and *CD73* ^−/−^ mice were administered NECA, a high‐affinity adenosine receptor enhancer, in an acute pancreatitis model (Figure 7A). We assessed tissue injury in H&E staining and found *CD73* ^−/−^ mice demonstrate significantly less pancreatitis area per field compared to their caerulein‐only‐treated genotype comparison (*p* < .01) (Figure 7B,C). To evaluate the effect of enhanced adenosine signaling on metaplasia pancreatitis area, an IHC stain for Cytokeratin‐19 was conducted to investigate the amount of metaplastic ductal cells present at Day 1 in NECA‐treated wild‐type and *CD73* ^−/−^ mice. Our analysis revealed NECA‐treated *CD73* ^−/−^ mice present significantly decreased amount of metaplasia compared to the caerulein‐only‐treated *CD73* ^−/−^ mice (*p* < .05) (Figure 7D,E). In contrast, there were similar levels of Cytokeratin‐19‐positive cells in NECA‐treated and caerulein‐only‐treated wild‐type mice at Day 1 (*p* = n.s.), suggesting that the most significant therapeutic effect was seen in animals that were previously adenosine depleted. Similarly, when MPO protein expression was evaluated by IHC (Figure 7F,G), we observed increased levels in *CD73* ^−/−^ pancreata compared to WT mice after caerulein‐only treatment; however, NECA administration promoted a markedly decrease in MPO levels in *CD73* ^−/−^ pancreata when compared with caerulein‐only *CD73* ^−/−^ treated mice only, suggesting both neutrophils and MPO secretion may participate in the metaplastic process during an acute pancreatitis setting.

## 4 DISCUSSION

Acinar and ductal cells comprise the exocrine component of pancreatic parenchymal function. Acinar cells are highly specialized cells characterized by zymogen granules and abundant rough endoplasmic reticulum. Acinar cells are responsible for synthesizing, storing, and secreting digestive enzymes including amylase, lipase, peptidase, and nucleases. In addition, acinar cells are the major source of trypsinogen, a component of pancreatic juice that is cleaved to trypsin by enteropeptidases in the intestinal mucosa.^73^, ^74^ The main function of the pancreatic ducts is to carry fluid containing digestive enzymes secreted from acinar cells. Ductal cells are a major source of sodium bicarbonate (NaHCO_3_) rich fluid which flushes out and neutralizes pH from digestive enzymes.^75^ In pancreatitis, the ductal epithelium does not appropriately regulate pH and more neutral pH or acidic pH is generated, causing obstruction and dilation of the duct lumen.^76^ The ductal system is intricate, with many small peripheral ducts all channeling to the main pancreatic duct. When gallstones, calcification or intraductal lesions block the exocrine component of the pancreas, patients develop pain and inflammation as there is an abnormal release of digestive enzymes, which can contribute to pancreatic fibrosis, calcification, and downstream pathophysiology. Neutrophil infiltration is one of the first pathogenic responses in early phases of pancreatitis^77^ and neutrophil accumulation is thought to prematurely activate trypsinogen release and aid in progression from acute to severe acute and chronic pancreatitis through production of reactive oxygen species (ROS) and hydrolases (reviewed in Ref. [77]). Neutrophil depletion significantly reduces serum amylase and reduces pancreatic injury in models of severe acute pancreatitis.^78^, ^79^, ^80^ The coordinated role of innate immune cells, including macrophages, and intrapancreatic cytokines and chemokines in pancreatic injury is important for resolution of injury and restoration of organ function.

In this manuscript, we describe that ductal cells and possibly subsets of ADM can express CD73 in the context of caerulein‐mediated acute or chronic pancreatitis. Through analysis of published single nuclear or single‐cell RNA‐seq datasets and immunohistochemistry, we show ductal cells and immune cells within interstitial spaces that express CD73 and we demonstrate acinar‐to‐ductal metaplasia may be a CD73‐mediated reparative process in the pancreas. We show CD73 promotes adenosine generation, a critical nucleoside to resolve tissue injury in response to pancreatitis, and suggest the increased acinar‐to‐ductal metaplastic cells seen in *CD73* ^−/−^ mice during acute pancreatitis may arise as an attempted tissue repair process. Under chronic conditions, we observed the expression of CD39 in vivo on macrophages which may indicate that the innate immune system is contributing to sustained injury, while the relatively high expression of CD73 in vivo on immune cells including T cells may indicate a divergent immune‐mediated mechanism is contributing to CD73‐mediated tissue injury resolution. Future studies delineating the role of specific adenosine receptors in stromal and immune cells in pancreatitis models will establish the mechanistic consequences of elevated intrapancreatic adenosine on development and resolution of pancreatitis.

Sustained enzymatic activity of CD39 in *CD73* ^−/−^ mice implicates enhanced AMP and loss of extracellular adenosine which contribute to enhanced disease severity in pancreatitis. Extracellular AMP is necessary for the initial response to acute injury in the pancreas by promoting inflammation, as seen by similar levels of CD39 in both wild‐type and *CD73* ^−/−^ mice at Day 1. However, sustained AMP levels promote a more severe phenotype. Thus, after initial insult, in wild‐type mice at Day 4, CD73 enzymatic activity is increased to promote adenosine generation and signaling through adenosine receptors, which promotes inflammation resolution. However, in *CD73* ^−/−^ mice, sustained CD39 activity at Day 4 demonstrates reduced capacity to generate extracellular adenosine in the absence of CD73. This switch in modulation is also seen by HPLC in wild‐type mice by the upregulation of purinergic signaling at initial tissue injury and then significant downregulation of AMP levels at Day 4 while simultaneously promoting adenosine generation after sustained tissue injury to promote resolution.

While both genotypes showed similar tissue injury and histologic change at Day 1 in the acute pancreatitis model, by Day 4 the wild‐type mice showed a near complete resolution of inflammation, while the *CD73* ^−/−^ mice demonstrated significant residual pancreatitis injury. The increased presence of acinar‐to‐ductal metaplastic cells at Day 4 in *CD73* ^−/−^ mice compared to the wild type suggests that without adenosine generation, reparative processes are still necessary to mitigate pancreatic injury. Additionally, increased staining of Cytokeratin‐19 in pancreatitis areas compared to normal histologic areas in both wild type and *CD73* ^−/−^ mice, but even greater still in *CD73* ^−/−^ mice, demonstrates the response to injury is regulated by adenosine receptor activation. This suggests the enhanced CD73 activity partially by ductal cells or ADM and most prominently by extra‐epithelial cells in wild‐type mice is allowing for an immune‐mediated rapid resolution of pancreatitis. However, at Day 7 in *CD73* ^−/−^ mice there is also a near complete resolution of tissue injury. In addition, we observe by HPLC adenosine in *CD73* ^−/−^ mice at Day 4 indicating intracellular conversion of AMP to adenosine and subsequent transport of adenosine into the microenvironment by ENT1 transporters may have been mechanistically why *CD73* ^−/−^ mice were able to resolve caerulein‐induced pancreatitis by Day 7. Further studies will be required to investigate cellularity of adenosine receptor expression to determine how adenosine specifically reduces pancreatic inflammation.

In these experiments, in addition to histological changes and differences in ADM abundance, we quantified a significant increase in MPO+ Ly6G+ and Granzyme B+ cells in *CD73* ^−/−^ mice at Day 4, which demonstrates a potent pro‐inflammatory response in the absence of adenosine in response to caerulein‐induced injury. The binding of ATP to the P2X and P2Y families of receptors, expressed on neutrophils in humans and in vivo,^59^ enhances neutrophil phagocytosis, chemotaxis, and oxidative burst. Under conditions of enhanced purinergic signaling, the resulting sustained activity and chemotactic ability of neutrophils promote sustained tissue injury and slower inflammation resolution. We experimentally show the significant impact of neutrophils in our genetic model using neutrophil deletion experiments which significantly reduced inflammation in *CD73* ^−/−^ mice. In addition, in NECA‐treated mice, we predict the high adenosine concentration at Day 1 potentially promotes adenosine‐dependent activation of A_2A_, A_2B_, and A_3_ adenosine receptors over the high‐affinity A_1_ receptor to promote tissue regeneration and restrain MPO accumulation and metaplasia in acute pancreatitis.^44^, ^55^ This identifies adenosine receptor enhancers as potential therapeutic targets for patients with acute pancreatitis as a mechanism to rapidly eliminate persistent inflammation via activation of A_2_ and A_3_ receptors.

## AUTHOR CONTRIBUTIONS

Conceptualization: Jennifer M. Bailey‐Lundberg, Baylee J. O'Brien, Erika Y. Faraoni, Kathleen E. DelGiorno; Data Curation: Baylee J. O'Brien, Lincoln N. Strickland, Erika Y. Faraoni, Tingting Mills, Samantha Mota, Victoria Mota, Xuebo Chen; Formal analysis: Baylee J. O'Brien, Erika Y. Faraoni, Kathleen E. DelGiorno, Jennifer M. Bailey‐Lundberg; Writing—Original Draft: Baylee J. O'Brien; Writing—Review and Editing: Erika Y. Faraoni, Jennifer M. Bailey‐Lundberg, Kathleen E. DelGiorno; Resources and Funding acquisition: Jennifer M. Bailey‐Lundberg, Holger K. Eltzschig, Kathleen E. DelGiorno.

## FUNDING INFORMATION

Texas Medical Center Digestive Disease Center Pilot and Feasibility Award NIH‐NIDDK‐2P30 056338‐16 and R21CA249924‐01 (J.M.B‐L). National Institute of Health Grants R01HL154720, R01DK122796, R01HL133900 and Department of Defense Grant W81XWH2110032 to H.K.E. Vanderbilt Digestive Disease Research Center Pilot and Feasibility Grant (NIH‐NIDDK P30 058404), American Gastroenterological Association Research Scholar Award (AGA2021‐13‐02), and NIH‐NIGMS R35 GM142709 (K.E.D.).

## DISCLOSURES

The authors have no conflicts of interest to disclose.

## Data Availability

The data, analytic methods, and study materials are stored on a University Lab Archives account and are available upon request.

## References

[1] Yadav D , Lowenfels AB . Trends in the epidemiology of the first attack of acute pancreatitis: a systematic review. Pancreas. 2006;33(4):323‐330. doi:10.1097/01.mpa.0000236733.31617.52 17079934

[2] Lerch MM , Gorelick FS . Models of acute and chronic pancreatitis. Gastroenterology. 2013;144(6):1180‐1193. doi:10.1053/j.gastro.2012.12.043 23622127

[3] Scherer J , Singh VP , Pitchumoni CS , Yadav D . Issues in hypertriglyceridemic pancreatitis: an update. J Clin Gastroenterol. 2014;48(3):195‐203. doi:10.1097/01.mcg.0000436438.60145.5a 24172179 PMC3939000

[4] Sonnenday CJ . Disorders of the exocrine pancreas. In: Hammer GD , McPhee SJ , eds. Pathophysiology of Disease: An Introduction to Clinical Medicine. McGraw‐Hill Education; 2013:784.

[5] Sattin A , Rall TW . The effect of adenosine and adenine nucleotides on the cyclic adenosine 3′, 5′‐phosphate content of guinea pig cerebral cortex slices. Mol Pharmacol. 1970;6(1):13‐23. 4354003

[6] Drury AN , Szent‐Györgyi A . The physiological activity of adenine compounds with especial reference to their action upon the mammalian heart. J Physiol. 1929;68(3):213‐237. doi:10.1113/jphysiol.1929.sp002608 16994064 PMC1402863

[7] Leone RD , Emens LA . Targeting adenosine for cancer immunotherapy. J Immunother Cancer. 2018;6(1):57. doi:10.1186/s40425-018-0360-8 29914571 PMC6006764

[8] King RJ , Shukla SK , He C , et al. CD73 induces GM‐CSF/MDSC‐mediated suppression of T cells to accelerate pancreatic cancer pathogenesis. Oncogene. 2022;41(7):971‐982. doi:10.1038/s41388-021-02132-6 35001076 PMC8840971

[9] Zhao J , Soto LMS , Wang H , et al. Overexpression of CD73 in pancreatic ductal adenocarcinoma is associated with immunosuppressive tumor microenvironment and poor survival. Pancreatology. 2021;21:942‐949. doi:10.1016/j.pan.2021.03.018 33832821 PMC8802341

[10] Singh K , Faraoni E , Dai Y , et al. Pancreatic Cancer Ductal Cell of Origin Drives CD73‐Dependent Generation of Immunosuppressive Adenosine. CellPress Sneak Peek (SSRN); 2021.

[11] Chen Q , Pu N , Yin H , et al. CD73 acts as a prognostic biomarker and promotes progression and immune escape in pancreatic cancer. J Cell Mol Med. 2020;24(15):8674‐8686. doi:10.1111/jcmm.15500 32643277 PMC7412695

[12] Shevchenko I , Mathes A , Groth C , et al. Enhanced expression of CD39 and CD73 on T cells in the regulation of anti‐tumor immune responses. Onco Targets Ther. 2020;9(1):1744946. doi:10.1080/2162402X.2020.1744946 PMC7790505 33457090

[13] Ma XL , Shen MN , Hu B , et al. CD73 promotes hepatocellular carcinoma progression and metastasis via activating PI3K/AKT signaling by inducing Rap1‐mediated membrane localization of P110β and predicts poor prognosis. J Hematol Oncol. 2019;12(1):37. doi:10.1186/s13045-019-0724-7 30971294 PMC6458749

[14] Arab S , Hadjati J . Adenosine blockage in tumor microenvironment and improvement of cancer immunotherapy. Immune Netw. 2019;19(4):e23. doi:10.4110/in.2019.19.e23 31501711 PMC6722273

[15] Beavis PA , Stagg J , Darcy PK , Smyth MJ . CD73: a potent suppressor of antitumor immune responses. Trends Immunol. 2012;33(5):231‐237. doi:10.1016/j.it.2012.02.009 22487321

[16] Antonioli L , Fornai M , Pellegrini C , et al. Adenosine signaling in the tumor microenvironment. Adv Exp Med Biol. 2021;1270:145‐167. doi:10.1007/978-3-030-47189-7_9 33123998

[17] Faraoni EY , Strickland LN , O'Brien BJ , et al. Radiofrequency ablation in combination with CD73 inhibitor AB680 reduces tumor growth and enhances anti‐tumor immunity in a syngeneic model of pancreatic ductal adenocarcinoma. Front Oncol. 2022;12:995027. doi:10.3389/fonc.2022.995027 36147911 PMC9486545

[18] Harvey JB , Phan LH , Villarreal OE , Bowser JL . CD73's potential as an immunotherapy target in gastrointestinal cancers. Front Immunol. 2020;11:508. doi:10.3389/fimmu.2020.00508 32351498 PMC7174602

[19] Luo F , Le NB , Mills T , et al. Extracellular adenosine levels are associated with the progression and exacerbation of pulmonary fibrosis. FASEB J. 2016;30(2):874‐883. doi:10.1096/fj.15-274845 26527068 PMC4714555

[20] Karmouty‐Quintana H , Philip K , Acero LF , et al. Deletion of ADORA2B from myeloid cells dampens lung fibrosis and pulmonary hypertension. FASEB J. 2015;29(1):50‐60. doi:10.1096/fj.14-260182 25318478 PMC4763976

[21] Burnstock G , Vaughn B , Robson SC . Purinergic signalling in the liver in health and disease. Purinergic Signal. 2014;10(1):51‐70. doi:10.1007/s11302-013-9398-8 24271096 PMC3944046

[22] Burnstock G , Novak I . Purinergic signalling in the pancreas in health and disease. J Endocrinol. 2012;213(2):123‐141. doi:10.1530/JOE-11-0434 22396456

[23] Imarisio C , Alchera E , Sutti S , et al. Adenosine a(2a) receptor stimulation prevents hepatocyte lipotoxicity and non‐alcoholic steatohepatitis (NASH) in rats. Clin Sci (Lond). 2012;123(5):323‐332. doi:10.1042/CS20110504 22439844

[24] Karmouty‐Quintana H , Zhong H , Acero L , et al. The A_2B_ adenosine receptor modulates pulmonary hypertension associated with interstitial lung disease. FASEB J. 2012;26(6):2546‐2557. doi:10.1096/fj.11-200907 22415303 PMC3650483

[25] Zhou Y , Schneider DJ , Morschl E , et al. Distinct roles for the A_2B_ adenosine receptor in acute and chronic stages of bleomycin‐induced lung injury. J Immunol. 2011;186(2):1097‐1106. doi:10.4049/jimmunol.1002907 21149612 PMC3607290

[26] Peng Z , Fernandez P , Wilder T , et al. Ecto‐5′‐nucleotidase (CD73) ‐mediated extracellular adenosine production plays a critical role in hepatic fibrosis. FASEB J. 2008;22(7):2263‐2272. doi:10.1096/fj.07-100685 18263696

[27] Montesinos MC , Gadangi P , Longaker M , et al. Wound healing is accelerated by agonists of adenosine A2 (G alpha s‐linked) receptors. J Exp Med. 1997;186(9):1615‐1620. doi:10.1084/jem.186.9.1615 9348321 PMC2199104

[28] Eckle T , Kewley EM , Brodsky KS , et al. Identification of hypoxia‐inducible factor HIF‐1A as transcriptional regulator of the A_2B_ adenosine receptor during acute lung injury. J Immunol. 2014;192(3):1249‐1256. doi:10.4049/jimmunol.1100593 24391213 PMC3946986

[29] Eltzschig HK , Sitkovsky MV , Robson SC . Purinergic signaling during inflammation. N Engl J Med. 2012;367(24):2322‐2333. doi:10.1056/NEJMra1205750 23234515 PMC3675791

[30] Dixit A , Cheema H , George J , et al. Extracellular release of ATP promotes systemic inflammation during acute pancreatitis. Am J Physiol Gastrointest Liver Physiol. 2019;317(4):G463‐G475. doi:10.1152/ajpgi.00395.2018 31433214 PMC6842987

[31] de Leve S , Wirsdörfer F , Jendrossek V . Targeting the immunomodulatory CD73/adenosine system to improve the therapeutic gain of radiotherapy. Front Immunol. 2019;10:698. doi:10.3389/fimmu.2019.00698 31024543 PMC6460721

[32] Antonioli L , Pacher P , Vizi ES , Haskó G . CD39 and CD73 in immunity and inflammation. Trends Mol Med. 2013;19(6):355‐367. doi:10.1016/j.molmed.2013.03.005 23601906 PMC3674206

[33] Allard B , Allard D , Buisseret L , Stagg J . The adenosine pathway in immuno‐oncology. Nat Rev Clin Oncol. 2020;17(10):611‐629. doi:10.1038/s41571-020-0382-2 32514148

[34] Toldo S , Zhong H , Mezzaroma E , et al. GS‐6201, a selective blocker of the A_2B_ adenosine receptor, attenuates cardiac remodeling after acute myocardial infarction in the mouse. J Pharmacol Exp Ther. 2012;343(3):587‐595. doi:10.1124/jpet.111.191288 22923737 PMC11047795

[35] Chan ES , Cronstein BN . Adenosine in fibrosis. Mod Rheumatol. 2010;20(2):114‐122. doi:10.1007/s10165-009-0251-4 19949965 PMC3129242

[36] Bekisz JM , Lopez CD , Corciulo C , et al. The role of adenosine receptor activation in attenuating cartilaginous inflammation. Inflammation. 2018;41(4):1135‐1141. doi:10.1007/s10753-018-0781-z 29656316

[37] Bono MR , Fernández D , Flores‐Santibáñez F , Rosemblatt M , Sauma D . CD73 and CD39 ectonucleotidases in T cell differentiation: beyond immunosuppression. FEBS Lett. 2015;589(22):3454‐3460. doi:10.1016/j.febslet.2015.07.027 26226423

[38] Regateiro FS , Cobbold SP , Waldmann H . CD73 and adenosine generation in the creation of regulatory microenvironments. Clin Exp Immunol. 2013;171(1):1‐7. doi:10.1111/j.1365-2249.2012.04623.x 23199317 PMC3530089

[39] Kubersky SM , Hirschhorn R , Broekman MJ , Cronstein BN . Occupancy of adenosine receptors on human neutrophils inhibits respiratory burst stimulated by ingestion of complement‐coated particles and occupancy of chemoattractant but not Fc receptors. Inflammation. 1989;13(5):591‐599. doi:10.1007/BF00916765 2807522

[40] Ehrentraut H , Clambey ET , McNamee EN , et al. CD73+ regulatory T cells contribute to adenosine‐mediated resolution of acute lung injury. FASEB J. 2013;27(6):2207‐2219. doi:10.1096/fj.12-225201 23413361 PMC3659359

[41] Granja T , Körner A , Glück C , et al. Targeting CD39 toward activated platelets reduces systemic inflammation and improves survival in sepsis: a preclinical pilot study. Crit Care Med. 2019;47(5):e420‐e427. doi:10.1097/CCM.0000000000003682 30730441

[42] Ramadan A , Naydenova Z , Stevanovic K , Rose JB , Coe IR . The adenosine transporter, ENT1, in cardiomyocytes is sensitive to inhibition by ethanol in a kinase‐dependent manner: implications for ethanol‐dependent cardioprotection and nucleoside analog drug cytotoxicity. Purinergic Signal. 2014;10(2):305‐312. doi:10.1007/s11302-013-9391-2 24163005 PMC4040176

[43] Morote‐Garcia JC , Köhler D , Roth JM , et al. Repression of the equilibrative nucleoside transporters dampens inflammatory lung injury. Am J Respir Cell Mol Biol. 2013;49(2):296‐305. doi:10.1165/rcmb.2012-0457OCx 23590299 PMC5455298

[44] Barletta KE , Ley K , Mehrad B . Regulation of neutrophil function by adenosine. Arterioscler Thromb Vasc Biol. 2012;32(4):856‐864. doi:10.1161/ATVBAHA.111.226845 22423037 PMC3353547

[45] Manjunath S , Sakhare PM . Adenosine and adenosine receptors: newer therapeutic perspective. Indian J Pharmacol. 2009;41(3):97‐105. doi:10.4103/0253-7613.55202 20442815 PMC2861820

[46] Fredholm BB , IJzerman AP , Jacobson KA , Linden J , Müller CE . International Union of Basic and Clinical Pharmacology. LXXXI. Nomenclature and classification of adenosine receptors—an update. Pharmacol Rev. 2011;63(1):1‐34. doi:10.1124/pr.110.003285 21303899 PMC3061413

[47] Olah ME , Stiles GL . Adenosine receptor subtypes: characterization and therapeutic regulation. Annu Rev Pharmacol Toxicol. 1995;35:581‐606. doi:10.1146/annurev.pa.35.040195.003053 7598508

[48] Wang J , Miao Y . Mechanistic insights into specific G protein interactions with adenosine receptors. J Phys Chem B. 2019;123(30):6462‐6473. doi:10.1021/acs.jpcb.9b04867 31283874 PMC7026936

[49] Schulte G , Fredholm BB . Signalling from adenosine receptors to mitogen‐activated protein kinases. Cell Signal. 2003;15(9):813‐827. doi:10.1016/s0898-6568(03)00058-5 12834807

[50] Ohta A , Sitkovsky M . Role of G‐protein‐coupled adenosine receptors in downregulation of inflammation and protection from tissue damage. Nature. 2001;414(6866):916‐920. doi:10.1038/414916a 11780065

[51] Faraoni EY , Ju C , Robson SC , Eltzschig HK , Bailey‐Lundberg JM . Purinergic and adenosinergic signaling in pancreatobiliary diseases. Front Physiol. 2022;13:849258. doi:10.3389/fphys.2022.849258 35360246 PMC8964054

[52] Gessi S , Merighi S , Varani K , Leung E , Mac Lennan S , Borea PA . The A3 adenosine receptor: an enigmatic player in cell biology. Pharmacol Ther. 2008;117(1):123‐140. doi:10.1016/j.pharmthera.2007.09.002 18029023

[53] Szabó C , Scott GS , Virág L , et al. Suppression of macrophage inflammatory protein (MIP)‐1alpha production and collagen‐induced arthritis by adenosine receptor agonists. Br J Pharmacol. 1998;125(2):379‐387. doi:10.1038/sj.bjp.0702040 9786512 PMC1565610

[54] Mabley J , Soriano F , Pacher P , et al. The adenosine A3 receptor agonist, N6‐(3‐iodobenzyl)‐adenosine‐5′‐N‐methyluronamide, is protective in two murine models of colitis. Eur J Pharmacol. 2003;466(3):323‐329. doi:10.1016/s0014-2999(03)01570-x 12694816

[55] Truong LD , Trostel J , McMahan R , Chen JF , Garcia GE . Macrophage A_2A_ adenosine receptors are essential to protect from progressive kidney injury. Am J Pathol. 2016;186(10):2601‐2613. doi:10.1016/j.ajpath.2016.06.017 27520357 PMC5222981

[56] Junger WG . Immune cell regulation by autocrine purinergic signalling. Nat Rev Immunol. 2011;11(3):201‐212. doi:10.1038/nri2938 21331080 PMC4209705

[57] Chen NM , Singh G , Koenig A , et al. NFATc1 links EGFR signaling to induction of Sox9 transcription and acinar‐ductal transdifferentiation in the pancreas. Gastroenterology. 2015;148(5):1024‐1034.e9. doi:10.1053/j.gastro.2015.01.033 25623042 PMC4409493

[58] Csóka B , Törő G , Vindeirinho J , et al. A_2A_ adenosine receptors control pancreatic dysfunction in high‐fat‐diet‐induced obesity. FASEB J. 2017;31(11):4985‐4997. doi:10.1096/fj.201700398R 28765173 PMC5636705

[59] Wang X , Chen D . Purinergic regulation of neutrophil function. Front Immunol. 2018;9:399. doi:10.3389/fimmu.2018.00399 29545806 PMC5837999

[60] Wang L , Xie D , Wei D . Pancreatic acinar‐to‐ductal metaplasia and pancreatic cancer. Methods Mol Biol. 2019;1882:299‐308. doi:10.1007/978-1-4939-8879-2_26 30378064

[61] Del Poggetto E , Ho IL , Balestrieri C , et al. Epithelial memory of inflammation limits tissue damage while promoting pancreatic tumorigenesis. Science. 2021;373(6561):eabj0486. doi:10.1126/science.abj0486 34529467 PMC9733946

[62] Strobel O , Dor Y , Alsina J , et al. In vivo lineage tracing defines the role of acinar‐to‐ductal transdifferentiation in inflammatory ductal metaplasia. Gastroenterology. 2007;133(6):1999‐2009. doi:10.1053/j.gastro.2007.09.009 18054571 PMC2254582

[63] Means AL , Logsdon CD . Acinar ductal metaplasia: yap fills a gap. Gastroenterology. 2016;151(3):393‐395. doi:10.1053/j.gastro.2016.07.022 27456389

[64] Liou GY , Döppler H , Necela B , et al. Macrophage‐secreted cytokines drive pancreatic acinar‐to‐ductal metaplasia through NF‐κB and MMPs. J Cell Biol. 2013;202(3):563‐577. doi:10.1083/jcb.201301001 23918941 PMC3734091

[65] Ma Z , Lytle NK , Chen B , et al. Single‐cell transcriptomics reveals a conserved metaplasia program in pancreatic injury. Gastroenterology. 2022;162(2):604‐620.e20. doi:10.1053/j.gastro.2021.10.027 34695382 PMC8792222

[66] Tosti L , Hang Y , Debnath O , et al. Single nucleus and in situ RNA sequencing reveals cell topographies in the human pancreas. Gastroenterology. 2020;160:1330‐1344.e11. doi:10.1053/j.gastro.2020.11.010 33212097

[67] Stuart T , Butler A , Hoffman P , et al. Comprehensive integration of single‐cell data. Cell. 2019;177(7):1888‐1902.e21. doi:10.1016/j.cell.2019.05.031 31178118 PMC6687398

[68] Kittel A , Garrido M , Varga G . Localization of NTPDase1/CD39 in normal and transformed human pancreas. J Histochem Cytochem. 2002;50(4):549‐556. doi:10.1177/002215540205000412 11897808

[69] Kittel A , Pelletier J , Bigonnesse F , et al. Localization of nucleoside triphosphate diphosphohydrolase‐1 (NTPDase1) and NTPDase2 in pancreas and salivary gland. J Histochem Cytochem. 2004;52(7):861‐871. doi:10.1369/jhc.3A6167.2004 15208353

[70] Zhao Z , Liu W . Pancreatic cancer: a review of risk factors, diagnosis, and treatment. Technol Cancer Res Treat. 2020;19:1533033820962117. doi:10.1177/1533033820962117 33357065 PMC7768873

[71] Garcia PE , Scales MK , Allen BL , Pasca di Magliano M . Pancreatic fibroblast heterogeneity: from development to cancer. Cells. 2020;9(11):2464. doi:10.3390/cells9112464 33198201 PMC7698149

[72] Hendley AM , Wang YJ , Polireddy K , et al. p120 catenin suppresses basal epithelial cell extrusion in invasive pancreatic neoplasia. Cancer Res. 2016;76(11):3351‐3363. doi:10.1158/0008-5472.CAN-15-2268 27032419 PMC4891257

[73] Lugea A , Waldron RT , Mareninova OA , et al. Human pancreatic acinar cells: proteomic characterization, physiologic responses, and organellar disorders in ex vivo pancreatitis. Am J Pathol. 2017;187(12):2726‐2743. doi:10.1016/j.ajpath.2017.08.017 28935577 PMC5718097

[74] Das SL , Kennedy JI , Murphy R , Phillips AR , Windsor JA , Petrov MS . Relationship between the exocrine and endocrine pancreas after acute pancreatitis. World J Gastroenterol. 2014;20(45):17196‐17205. doi:10.3748/wjg.v20.i45.17196 25493036 PMC4258592

[75] Grapin‐Botton A . Ductal cells of the pancreas. Int J Biochem Cell Biol. 2005;37(3):504‐510. doi:10.1016/j.biocel.2004.07.010 15618005

[76] Ishiguro H , Yamamoto A , Nakakuki M , et al. Physiology and pathophysiology of bicarbonate secretion by pancreatic duct epithelium. Nagoya J Med Sci. 2012;74(1–2):1‐18. 22515107 PMC4831246

[77] Wan J , Ren Y , Yang X , Li X , Xia L , Lu N . The role of neutrophils and neutrophil extracellular traps in acute pancreatitis. Front Cell Dev Biol. 2020;8:565758. doi:10.3389/fcell.2020.565758 33553136 PMC7859271

[78] Folias AE , Penaranda C , Su AL , Bluestone JA , Hebrok M . Aberrant innate immune activation following tissue injury impairs pancreatic regeneration. PLoS ONE. 2014;9(7):e102125. doi:10.1371/journal.pone.0102125 25010227 PMC4092101

[79] Inoue S , Nakao A , Kishimoto W , et al. Anti‐neutrophil antibody attenuates the severity of acute lung injury in rats with experimental acute pancreatitis. Arch Surg. 1995;130(1):93‐98. doi:10.1001/archsurg.1995.01430010095020 7802585

[80] John DS , Aschenbach J , Krüger B , et al. Deficiency of cathepsin C ameliorates severity of acute pancreatitis by reduction of neutrophil elastase activation and cleavage of E‐cadherin. J Biol Chem. 2019;294(2):697‐707. doi:10.1074/jbc.RA118.004376 30455353 PMC6333881

